# Measuring urbanicity as a risk factor for childhood wheeze in a transitional area of coastal ecuador: a cross-sectional analysis

**DOI:** 10.1136/bmjresp-2020-000679

**Published:** 2020-11-30

**Authors:** Alejandro Rodriguez, Laura Rodrigues, Martha Chico, Maritza Vaca, Mauricio Lima Barreto, Elizabeth Brickley, Philip J Cooper

**Affiliations:** 1 Facultad de ciencias Médicas de la salud y la Vida, Universidad Internacional del Ecuador, Quito, Pichincha, Ecuador; 2 SCAALA project, Fundacion Ecuatoriana para la Investigacion en Salud (FEPIS), Quininde, Esmeraldas, Ecuador; 3 Faculty of Epidemiology and Population Health, London School of Hygiene and Tropical Medicine, London, UK; 4 Centro de de Integração de Dados e Conhecimentos para Saúde (CIDACS) FIOCRUZ, Salvador, Bahia, Brazil; 5 Instituto de Saude Coletiva, Universidade Federal da Bahia, Salvador, Bahia, Brazil; 6 Faculty of Epidemiology and Population Health, London School of Hygiene & Tropical Medicine, London, UK; 7 Institute of Infection and Immunity, St George’s University of London, London, Esmeraldas, UK

**Keywords:** asthma, asthma epidemiology, paediatric asthma

## Abstract

**Background:**

The urbanisation process has been associated with increases in asthma prevalence, an observation supported largely by studies comparing urban with rural populations. The nature of this association remains poorly understood, likely because of the limitations of the urban–rural approach to understand what a multidimensional process is.

**Objective:**

This study explored the relationship between the urbanisation process and asthma prevalence using a multidimensional and quantitative measure of urbanicity.

**Methods:**

A cross-sectional analysis was conducted in 1843 children living in areas with diverse levels of urbanisation in the district of Quinindé, Ecuador in 2013–2015. Categorical principal components analysis was used to generate an urbanicity score derived from 18 indicators measured at census ward level based on data from the national census in 2010. Indicators represent demographic, socioeconomic, built environment and geographical dimensions of the urbanisation process. Geographical information system analysis was used to allocate observations and urban characteristics to census wards. Logistic random effects regression models were used to identify associations between urbanicity score, urban indicators and three widely used definitions for asthma.

**Results:**

The prevalence of wheeze ever, current wheeze and doctor diagnosis of asthma was 33.3%, 13% and 6.9%, respectively. The urbanicity score ranged 0–10. Positive significant associations were observed between the urbanicity score and wheeze ever (adjusted OR=1.033, 95% CI 1.01 to 1.07, p=0.05) and doctor diagnosis (adjusted OR=1.06, 95% CI 1.02 to 1.1, p=0.001). For each point of increase in urbanicity score, the prevalence of wheeze ever and doctor diagnosis of asthma increased by 3.3% and 6%, respectively. Variables related to socioeconomic and geographical dimensions of the urbanisation process were associated with greater prevalence of wheeze/asthma outcomes.

**Conclusions:**

Even small increases in urbanicity are associated with a higher prevalence of asthma in an area undergoing the urban transition. The use of a multidimensional urbanicity indicator has greater explanatory power than the widely used urban–rural dichotomy to improve our understanding of how the process of urbanisation affects the risk of asthma.

**Key messages**

- This paper addresses the question of why the prevalence of asthma is associated with the process of urbanisation in low-income and middle-income countries, particularly in Latin America.
- To date, most studies exploring the effects of urbanisation on asthma have used simple urban–rural dichotomies, that can only identify the totality of multiple and often counter-balancing effects acting on asthma. Such an approach does not allow us to consider the multifactorial dimensions of the urbanisation process and cannot identify specific factors or conditions associated with asthma risk.
- Our study introducing a multidimensional approach based on a composite measure of urbanicity using different urban indicators to evaluate the effects of urbanisation on asthma in transitional populations. Our analysis illustrates the benefits of using a multidimensional measure of urbanicity to study the epidemiology of asthma, allowing us to understand better which components may be most relevant, compared with more traditional urban–rural dichotomies. This study is of general interest to public health researchers, epidemiologists and social scientists.

## Introduction

Studies conducted in low/middle-income countries (LMICs) have consistently associated the urbanisation process with temporal and geographical trends of increasing asthma prevalence.^1 2^ These studies have shown that asthma prevalence is frequently higher in urban compared with rural settings, indicating urban residence to be a potential risk factor.^2 3^ However, it is not yet clear how urban residence increases asthma susceptibility or the mechanisms by which the urbanisation process affects asthma prevalence.

Numerous environmental, social and behavioural changes related to the urbanisation process have been associated with differences in asthma prevalence between urban and rural populations.^4^ Changes in diet, sedentarism, reduction in the frequency of infections, reduction in family size, use of antibiotics, increases in environmental pollution, migration, among others, have been identified as possible risk factors for asthma.^5–7^ However, the nature of these associations remains poorly understood. A potential mechanism to explain rural–urban gradients in asthma prevalence in LMICs has been provided by the hygiene hypothesis in which childhood exposures to infectious diseases and a wide diversity of micro-organisms in the environment associated with traditional rural lifestyles, are hypothesised to provide protection against asthma.^8^ For example, helminth infections, endemic among many populations living in rural regions of LMICs and which have allergy-modulating effects, have been proposed as an explanation for the lower prevalence of asthma and allergies in rural populations.^9^ However, the findings of studies investigating the effects of childhood infections and microbial diversity on asthma prevalence in LMICs have been far from conclusive^9 10^ and there is a need to identify other factors associated with the process of urbanisation to explain urban–rural differences in asthma prevalence.

The process of urbanisation has been generally defined by the proportion of the population living in cities or urban areas.^11^ However, the idea that urbanisation mostly affects populations living in cities is too simplistic a view of this process, inevitably reducing the concept to a phenomenon of population density. In broad terms, urbanisation is defined as the gradual process of becoming urban and includes higher concentrations of people in relatively small areas, but also population growth by migration and natural increase, improvements in built infrastructure and changes in social and economic activities.^12^ Although such a definition covers the multidimensional nature of the process, it also introduces a longitudinal perspective that is difficult to evaluate in cross-sectional studies. The use of the ‘urbanicity’ becomes relevant in this context to overcome the longitudinal problem, a concept which refers to the presence of conditions that are more common in urban areas than in non-urban areas at any given point in time.^12^

Our knowledge of the relationship between urbanisation and asthma in LMICs is derived from studies comparing urban and rural populations.^3^ Although these studies have provided valuable information about the burden of the disease and differences in risk factors between urban and rural populations, they cannot take into account the multifactorial dimensions of the urbanisation process or how the specific conditions of urban living affect the prevalence of asthma, especially in populations within which levels of urbanisation are highly variable. The aim of this study was: (1) develop a multidimensional quantitative score of urbanicity; (2) explore the associations between urbanicity score, urban indicators and different definitions of asthma; and (3) compare the urbanicity score with the urban–rural approach and geo-political divisions as a predictor of asthma prevalence.

## Methods

### Context of the study

We used data from a birth cohort (the Estudio eCUAtoriano del impacto de infecciones sobre Vacunas, Inmunidad y el Desarrollo de enfermedades Alergicas (ECUAVIDA) cohort) that followed 2404 newborns of mothers living within the District of Quininde, Esmeraldas Province, to 8 years of age. The ECUAVIDA study is a prospective cohort designed to investigate the impact of prenatal and postnatal exposures to soil-transmitted helminth parasites on the development of asthma and other allergic diseases.^9^ The study began recruiting participants in November 2006 and finished in December 2009. Detailed information has been collected for each child around the time of the birth, at 7 and 13 months and at 2-3-5 and 8 years. Data collected for children include: demographic, lifestyle, psychosocial and dietary factors; childhood morbidity and clinical outcomes. Clinical evaluations include: stool samples for parasites; blood samples for DNA, measurements of vaccine responses and other measures of immune function/inflammation, anthropometrics and allergen skin prick test reactivity. Data from this cohort are particularly well suited to study the effects of urbanisation on asthma because individual-level, household-level and census tract-level data are available for all study participants.

### Study area and population

**Figure 1:**
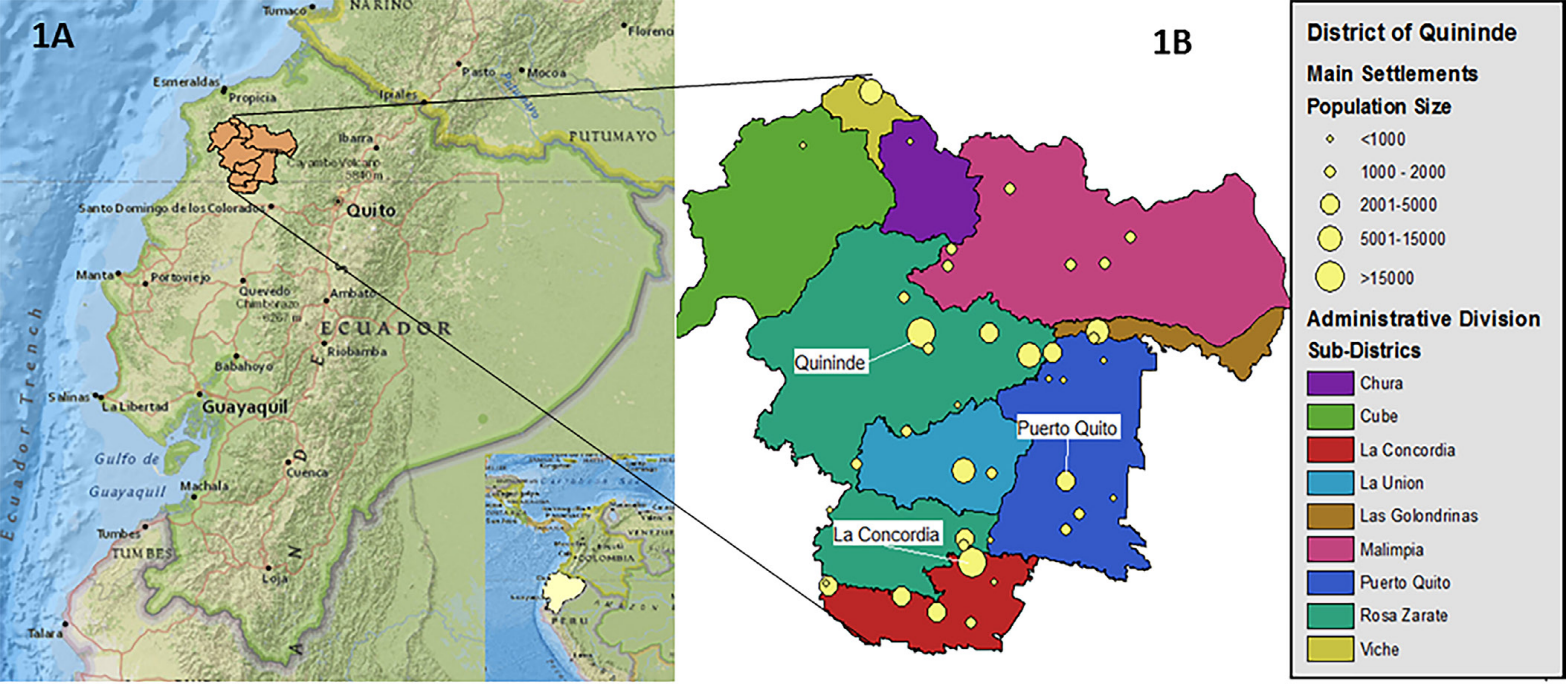
Study area: districts of Quininde and Puerto Quito and its political division. (A) Map of Ecuador and location of study area. (B) Geopolitical divisions of the study area by parish and main population settlements.

The study was conducted in the districts of Quinindé, located in northwest Ecuador, a transitional area where the population is undergoing a rapid transformation from a traditional rural to a more urban lifestyle. Although the recruitment of the cohort was defined by residence within the district of Quinindé, the district of Puerto Quito was included in the present analysis because of migration of cohort families to this district during follow-up. With an extension of 5019 km^2^, and approximately 200 000 inhabitants, the study area is divided into 9 subdistricts and 330 census wards, of which 38 represent settlements of different population sizes as district capitals, towns and communities (figure 1). The settlements of La Concordia, Quinindé and Puerto Quito are the only three census wards considered as urban areas by the National Institute of Statistical and Census of Ecuador (INEC).^13^ The main economic activities in this region are focused on cattle and agriculture, especially cultivation of African palm oil and tropical fruits. Provision of basic services and other facilities are present in larger settlements where coverage, however, is deficient.^13^

### Study design and sample

A cross-sectional analysis, nested within the birth cohort, was done to explore the effects of the urbanisation process on asthma prevalence. Asthma symptoms were measured at 5 years. Urban conditions were measured at the level of census wards using data from the last national census and obtained through INEC web page.^13^ The study population represented 77% of the 2404 newborns recruited into the ECUAVIDA birth cohort. Most children not included in the analysis had either migrated outside the study area (11%) or were lost to follow-up (12%) Geographical coordinates of the child’s household allowed each child to be referenced to a specific census ward.

### Data collection

#### Measures of urban conditions

Urbanisation is a highly complex process that affects all levels of human activity. No single discipline can fully describe the multidimensional nature of this process. Therefore, diverse approaches were necessary to define the different dimensions that comprises the urban process. Our study draws on several indicators used by urban geography, demography, urban health and sociological theories to define four dimensions of the urbanisation process.

Based on data available from the national census of 2010, 18 indicators were selected for inclusion in our urbanicity scale. These variables were classified into four groups representing some of the main dimensions of the urbanisation process (see table 1). (1) Demographic—representing the phenomenon of population concentration within restricted spaces (variables—population size and density). (2) Socioeconomic—representing changes in living conditions related to the rural–urban transition (non-agricultural activities, secondary education, commercial activities, housing constructed with cement, access to mobile phones, internet, computers and satellite tv). (3) Built environment—physical characteristics of the urban environment characterised by access to basic services, public and private institutions, and urban infrastructure (street paving, sewage access, electricity and educational and health institutions). (4) Geographical—representing spatial distribution of the settlement where the child lives (geopolitical division, proximity to urban centres and access to highways). Geographical information systems analysis was used to allocate observations and urban characteristics to respective census wards. Maps representing the urbanicity scale was built using ArcGIS V.10.2.2 (ESRI, California, USA).

**Table 1:** Urban indicators, definitions and summary statistics of the study area (n=330 wards)

Dimension/ indicators	Definitions	Scale/category	Summary statistics
**Demographic**			
Population size	The total number of people residing in a census ward.	Number of individuals	Mean=580 SD*=2360 Range=36–29 356
Population density	Number of people residing in a census ward divided by the land area of the same ward.	Number of individuals per km^2^	Mean=332 Range=1.5–4899 SD=924
**Socioeconomics**			
Non-agriculture activities	Number of people>18 years working in non-agricultural activities divided by the total number of people>18 years residing in a census ward.	Percentage	Mean=28.9 Range=0–98.2 SD=19.1
Secondary education	Number of people>18 years that have finished secondary education divided by the total number of people>18 years residing in a census ward.	Percentage	Mean=13 Range=0–43 SD=7.4
Commercial activities	Number of people>18 years working in commercial activities divided by the total number of people>18 years residing in a census ward.	Percentage	Mean=9 Range=0–52 SD=9.5
Concrete housing	Number of households building with iron roof and concrete walls and floors divided by the total number of households in a census ward.	Percentage	Mean=30 Range=0–77 SD=18
Mobile phone access	Number of households with access to mobile phones divided by the total number of households in a census ward.	Percentage	Mean=71 Range=19–97 SD=12.9
Internet access	Number of households with access to internet divided by the total number of households in a census ward.	Percentage	Mean=2 Range=0–13 SD=2.5
Computer access	Number of households with access to computers divided by the total number of households in a census ward.	Percentage	Mean=4 Range=0–28 SD=4.6
Satellite TV access	Number of households with access to satellite TV divided by the total number of households in a census ward.	Percentage	Mean=3 Range=0–66 SD=7.4
**Built environment**			
Pavement streets	Number of households with access to paved streets divided by the total number of households in a census ward.	Percentage	Mean=8 Range=0–78 SD=15.7
Sewage system	Number of households with access to sewage system divided by the total number of households in a census ward.	Percentage	Mean=2 Range=0–74 SD=7.2
Electricity	Number of households with access to the electricity grid divided by the total number of households in a census ward.	Percentage	Mean=78 Range=0–100 SD=22.6
Educational institutions	Number of educational institutions (primary and secondary education) present in a census ward.	Number of schools	Mean=1 Range=0–21 SD=2
Health facilities	Presence of health facilities in a census ward as health centres and hospitals.	None Health centres Hospital	n (%)=305 (92) n (%)=23(7) n (%)=2 (1)
Road connectivity	Presence of the National Highway in the ward.	No Yes	n (%)=221 (67) n (%)=109(33)
**Geographic**			
Geographical division	Geographical separations of the territory based on population settlement area and other sociodemographic characteristics. Wards divisions are delimited and classified by INEC.^13^	Countryside Community Town City	n (%)=292 (88) n (%)=30(9) n (%)=5 (2) n (%)=3 (1)
Urban closeness	Proximity of the wards with respect to cities. Classified as Urban, wards that contain cities; Periphery, wards located next to the cities; Distant, wards with no boundaries with the Cities.	Urban Periphery Distant	n (%)=4 (2) n (%)=55(17) n (%)=271 (81)

INEC, National Institute of Statistical and Census of Ecuador; SD, Standard Deviation.

#### Asthma definitions and asthma risk factors

Data on asthma symptoms and risk factors were collected between 2013 and 2015 using a questionnaire that included the core asthma questions of the International Study of Asthma and Allergies in Childhood.^14 15^ The questionnaire was administered to the child’s mother by a trained physician. Three different definitions were used for asthma: wheeze ever (Has your child ever had wheezing or whistling in the chest at any time in the past?), current wheeze (Has your child had wheezing or whistling in the chest in the past 12 months?) and doctor diagnosis of asthma (Has your child diagnosed of asthma by a doctor?) (table 2). Several asthma risk factors were included in our analyses as sex, maternal history of asthma, environmental tobacco smoke exposure in the child’s household, household overcrowding, dog inside the house, farm animals around the house, medical diagnostic of bronchitis, medical diagnostic of bronchopneumonia and atopy defined by the presence of allergen skin test reactivity at 5 years to any of the following aeroallergens: *Dermatophagoides pteronyssinus*/*farinae* mix, American cockroach (*Periplaneta americana*), fungi mix, dog, cat and mixed grass pollen (table 2).

**Table 2:** Asthma definitions and individual risk factors

Variables	Definitions	Categories	N (%)
Wheeze ever	Children who have ever had wheezing in life	No	1229 (66.7)
		Yes	614 (33.3)
		Missing	0
Current wheeze	Children who presented wheeze in the last 12 months	No	1603 (87)
		Yes	240 (13)
		Missing	2 (0.01)
Doctor diagnosis of asthma	Children who were diagnosed of asthma by a doctor	No	1715 (92.4)
		Yes	128 (6.9)
		Missing	12 (0.07)
Sex	Sex of the children	Female	900 (48.8)
		Male	943 (51.2)
		Missing	0
Maternal asthma	Children who have mothers with history of asthma	No	1698 (92.1)
		Yes	122 (6.6)
		Missing	23 (1.2)
Environmental tabaco Smoke	Smoking habit at home in the last 2 years by any of the family members	No	1599 (86.8)
		Yes	242 (13.1)
		Missing	2 (0.1)
Household overcrowding	Children living in houses with more than three people per bedroom	No	1369 (78.6)
		Yes	372 (21.4)
		Missing	102 (5.5%)
Dog at home	Children living in households with dogs inside the hose	No	1031 (55.9)
		Yes	812 (44.1)
		Missing	0
Farm animals	Children living in households with farm animals around the hose	No	565 (30.7)
		Yes	1277 (69.3)
		Missing	1 (0.1)
Bronchitis	Children who were diagnosed of bronchitis by a doctor	No	1559 (84.6)
		Yes	270 (14.7)
		Missing	14 (0.8)
Bronchopneumonia	Children who were diagnosed of bronchopneumonia by a doctor	No	1694 (92.8)
		Yes	131 (7.2)
		Missing	18 (1)
Atopy	Children who tested positive to skin prick test for	No	1571 (85.2)
		Yes	256 (13.9)
		Missing	16 (0.09)

### Statistical analyses

Categorical principal components analysis (CATPCA) was used to generate a composite measure of urbanicity using demographic, socioeconomic, built environment and geographical indicators. CATPCA is a multivariable data reduction method that summarises information from several correlated variables into one or more independent linear combinations representing most of the information from the original variables.^16^ In contrast to similar techniques that are restricted to numeric variables, CATPCA integrates quantitative and qualitative variables in the analysis assigning metric properties to each category of nominal or ordinal variables through optimal scaling.^17^ In CATPCA, the first component explains the highest proportion of observed variance while the second component accounts for most of the variance not explained by component 1, and so on. The original variables are associated with each component through component loadings that show contributions to a given component. Values of correlation range −1 to +1, with a larger absolute value indicating a stronger contribution of a variable to that component. Each component produces a Z score for each observation (in our case by each census ward) that summarises the contribution of all variables to each component.^16^

Based on a previous CATPCA analysis of urbanisation,^18^ which showed high component loadings and a high percentage of variance explained by the first component for urban indicators, we set our model to use the first component as a measure of urbanicity. To do that, we retained the minimum number of components allowed by the technique, or two components. The urbanicity score was interpreted such that higher Z values of the first component by census wards indicated a higher level of urbanicity. Subsequently, the urbanicity score was categorised in several groups representing diverse scales of urbanisation to assess its performance against other measures of urbanicity such as urban–rural classifications and political divisions.

Bivariate analyses using logistic random effects regression models were used to identify associations between asthma definitions, urbanicity score and urban indicators, allowing for two-level data structure (ie, at individual and census ward levels). Logistic random effects models are used to analyse multilevel data with a binary or ordinal outcome, in which the log odds of the outcomes are modelled as a linear combination of the predictor variables when data are clustered or there are both fixed and random effects.^19^ ORs were estimated for urbanicity score and asthma definitions adjusted for a priori confounders as sex, maternal asthma, environmental tabaco smoke, household overcrowding, dog at home, farm animals, bronchitis, bronchopneumonia and atopy. The urbanicity score was categorised in four levels (urbanicity scale) to be compared with other definitions of urbanicity using logistic regression. Additionally, univariate and multivariable logistic regression models were used to evaluate the associations between asthma definitions, urbanicity scale and other asthma risk factors. Multivariate models were selected using backwards stepwise regression and were those that explained the most variation in wheeze prevalence, those with the smallest mean square error, and the highest value of adjusted R^2^. Variables with p<0.05 were considered statistically significant. Statistical analyses were done using SPSS V.24 (IBM SPSS Statistic)

### Patient and public involvement

This research was done without patient involvement. Patients were not invited to comment on the study design and were not consulted to develop patient relevant outcomes or interpret the results. Patients were not invited to contribute to the writing or editing of this document for readability or accuracy.

## Results

### Urban indicators

We evaluated 1843 children living in 157 census wards of the total 330 wards present within the two study districts. Table 1 shows descriptive measures for 18 indicator variables representing demographic, socioeconomic, built environment and geographical dimensions for the 330 census wards that correspond to the districts of Quininde and Puerto Quito. There was considerable variation in indicator variables between census wards. The mean population by census wards was 580 inhabitants, ranging from 36 to 29 356, with an average population density of 332 people by km^2^. Approximately 71% of the working-age population was engaged in agricultural activities and only 13% of the population had received secondary education. Except for household electricity (78% coverage) and access to a mobile phone network (71%), household access to urban services was low.

### Asthma definitions and asthma risk factors

The prevalence of wheeze ever, current wheeze and doctor diagnosis was 33.3%, 13% and 6.9%, respectively (table 2). 51.2% of the children were males, 6.6% had history of maternal asthma, 13.1% presented environmental tabaco smoke, 21.4% of the children lived in overcrowded houses, 44% had a dog inside of the house and 69.3% of the study population had farm animals around the house. The prevalence of bronchitis, bronchopneumonia and atopy was 14.8%, 7.2% and 14%, respectively (table 2).

### Urbanicity score

**Figure 2:**
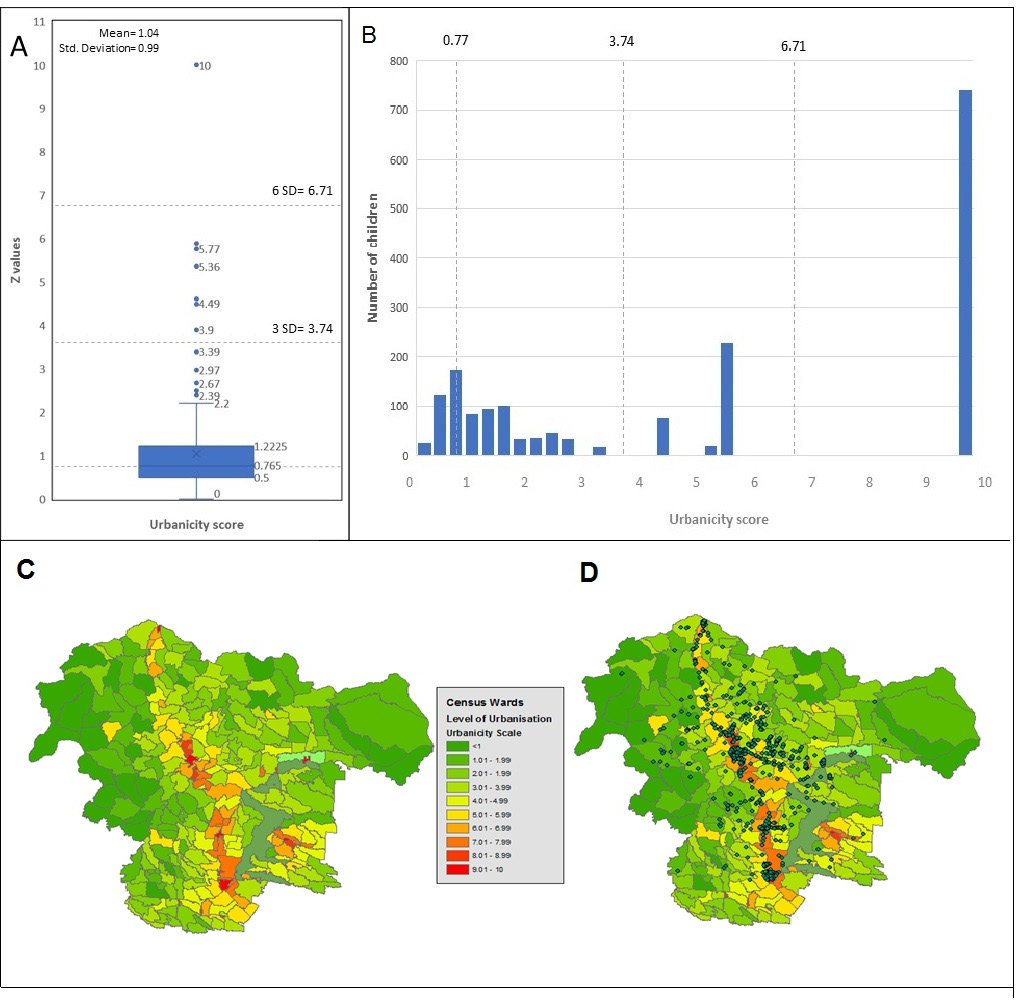
Urbanicity score: descriptive statistics and maps. (A) Box plot describes the distribution of the urbanicity score and segmented lines are cut points representing different levels of urbanicity: low urbanicity (wards with values less than the median 0.77), middle urbanicity (wards with values between 0.78 and 3.74), upper middle urbanicity (wards with values between 3.75 and 6.71) and high urbanicity (wards with values higher to 6.72). (B) Histogram showing the distribution on the children across the urbanicity score and the cut points of the urbanicity scale. (C) District of Quinindé showing urbanicity score by census ward. (D) District of Quinindé showing the geographical location of study households (green dots) within census wards.

Results of CATPCA analyses are provided in table 3. All 18 indicators were included in the model and all had positive loadings for the first component. Fourteen had loadings>0.5, 3 had loadings 0.4–0.5, and 1 (mobile phone access) had a low component loading <0.3). The total variance explained by the first component was 42.3%. The proportion of variance explained by each variable and the quantifications for each category for all variables are shown in online supplemental figure SF1 and online supplemental table ST1. To allow easier interpretation of the urbanicity score that was on a scale of 0–10 (0 being the least and 10 the most urban), 1.04 was added to each of the z values for the first component. Figure 2A shows a box plot with the distribution of the urbanicity score. A map was built to visualise the variation of the urbanicity score within the study area (figure 2C). For purposes of comparison with other urbanicity measures, the urbanicity score was categorise in four levels or scales based on its median and multiples of the SD (figure 2A). (1) Low urbanicity, wards with values less than the median (0.77); (2) middle urbanicity, wards with values between the median and 3 SD (0.78–3.74); (3) upper middle urbanicity, wards with values between 3 and 6 SDs (3.75–6.71); (4) high urbanicity, wards with values higher to 6 SDs (6.72). The locations of households by census wards are shown in figure 2D.

10.1136/bmjresp-2020-000679.supp1 Supplementary data

**Table 3:** Categorical principal components analysis: component loadings by demographic, socioeconomic, infrastructure and geographical indicators

Component loadings	Dimensions	
	1	2
Population size	0.782	−0.476
Population density	0.794	−0.384
Non-agriculture activities	0.654	−0.172
Secondary of education	0.803	0.250
Commercial activity	0.907	−0.161
Concrete housing	0.498	0.517
Mobile phone access	0.282	0.599
Internet access	0.644	0.231
Computer access	0.723	0.371
Satellite TV access	0.777	0.045
Pavement street	0.513	0.491
Sewage system	0.637	−0.244
Electricity	0.453	0.511
Educational Institutions	0.647	−0.499
Health facilities	0.587	−0.358
Road connectivity	0.404	0.493
Geographical division	0.740	−0.379
Urban closeness	0.519	0.273
Variance explained	**42.3%**	**15.1%**

Bold values represent the total variance accounted by the model, 57.4%

### Associations among urbanicity score, urban indicators and asthma

The results of bivariate analyses between urbanicity scores, urban indicators and asthma definitions are shown in table 4. Positive and significant associations were observed between the urbanicity score and wheeze ever (adjusted OR 1.033, 95% CI 1.0 to 1.07, p=0.05) and doctor diagnosis (adjusted OR 1.06, 95% CI 1.02 to 1.09, p=0.001). Thus, for a one-unit increase in urbanicity score, the risk of wheeze ever and doctor diagnosis increased by 3.3% and 6%, respectively. Associations between wheeze ever and urban indicators were observed for 3 of the 18 indicators: concrete housing (OR 1.12, 95% CI 1.03 to 1.20), p=0.008), sewage system (OR 1.12, 95% CI 1.03 to 1.21, p=0.005) and close access to highway (OR 1.41, 95% CI 1.05 to 1.88, p=0.021). An 10% increase in proportion of houses being constructed with concrete and of households with access to a sewage system were both associated with a 12% increased risk of wheeze. Children living in wards with close access to highways had 41% greater risk of wheeze compared with children living in wards without highway access. Two urban indicators were associated with current wheeze: adult secondary education levels (OR 1.15, 95% CI 1.0 to 1.30, p=0.04) and household computer (OR 1.25, 95% CI 1.01 to 1.44, p=0.014). Twelve indicators were associated with doctor diagnosis of asthma: population size (OR 1.14, 95% CI 1.04 to 1.24, p=0.006), secondary education (OR 1.18, 95% CI 1.05 to 1.31, p=0.006), commercial activities (OR 1.11, 95% CI 1.03 to 1.18, p=0.007), mobile phone access (OR 1.24, 95% CI 1.02 to 1.47, p=0.034), home internet access (OR 1.46, 95% CI 1.08 to 1.86, p=0.019), home computer access (OR 1.32, 95% CI 1.14 to 1.50, p=0.001), home satellite TV access (OR 1.08, 95% CI 1.04 to 1.13 p=0.001), household sewage systems (OR 1.16, 95% CI 1.02 to 1.29, p=0.02), presence of educational (OR 1.02, 95% CI 1.01 to 1.04, p=0.007) and health institutions (hospital vs none, OR 1.45, 95% CI 1.04 to 2.03, p=0.027), geopolitical division (town vs countryside, OR 1.46, 95% CI 1.03 to 2.07, p=0.034) and proximity to an urban centre (OR 0.62, 95% CI 0.43 to 0.87, p=0.006). Additionally, we conducted a bivariate analysis among atopy, urban score and urban indicators (online supplemental table 2). The analyses showed an inverse association among atopy, urbanicity score and urban indicators.

**Table 4:** Bivariate logistic regression among urbanicity score, urban variables and asthma definitions

Indicators	Wheeze ever			Current wheeze			Doctor diagnosis		
	OR	95% CI	P value	OR	95% CI	P value	OR	95% CI	P value
Urbanicity score ‡	1.033*	**(1.0 to 1.07)**	**0.05**	1.032†	(0.98 to 1.08)	0.156†	**1.06**	**(1.02 to 1.09)**	**0.001**
Population size§	1.04	(0.96 to 1.12)	0.356	0.96	(0.76 to 1.15)	0.660	**1.14**	**(1.04 to 1.24)**	**0.006**
Population density¶	1.02	(0.96 to 1.08)	0.533	0.98	(0.90 to 1.06)	0.702	1.07	(0.99 to 1.15)	0.082
Non-agriculture activities**	1.04	(0.99 to 1.07)	0.078	1.01	(0.95 to 1.07)	0.804	1.05	(0.99 to 1.12)	0.116
Secondary education**	1.10	(0.98 to 1.20)	0.09	**1.15**	**(1.0 to 1.30)**	**0.04**	**1.18**	**(1.05 to 1.31)**	**0.006**
Commercial activities**	1.06	(0.99 to 1.11)	0.057	1.05	(0.97 to 1.11)	0.221	**1.11**	**(1.03 to 1.18**)	**0.007**
Concrete housing**	**1.12**	**(1.03 to 1.20)**	**0.008**	1.10	(0.98 to 1.19)	0.1	1.15	(1.00 to 1.31)	0.054
Mobile phone access**	1.09	(0.96 to 1.22)	0.154	1.14	(0.95 to 1.32)	0.133	**1.24**	**(1.02 to 1.47)**	**0.034**
Internet access**	1.21	(0.92 to 1.49)	0.153	1.16	(0.76 to 1.57)	0.429	**1.46**	**(1.08 to 1.86)**	**0.019**
Computer access**	1.15	(0.99 to 1.32)	0.064	**1.25**	**(1.01 to 1.44)**	**0.014**	**1.32**	**(1.14 to 1.50)**	**0.001**
Satellite TV access**	1.04	(0.99 to 1.08)	0.109	1.06	(0.99 to 1.12)	0.055	**1.08**	**(1.04 to 1.13)**	**0.001**
Pavement street**	1.06	(0.98 to 1.13)	0.126	1.06	(0.96 to 1.14)	0.224	1.08	(0.96 to 1.19)	0.185
Sewage system**	**1.12**	(1.03 to 1.21)	**0.005**	1.03	(0.92 to 1.13)	0.614	**1.16**	**(1.02 to 1.29)**	**0.02**
Electricity**	1.01	(0.91 to 1.10)	0.843	1.13	(0.92 to 1.32)	0.22	1.20	(0.94 to 1.46)	0.137
Educational institutions	1.01	(0.92 to 1.17)	0.443	0.99	(0.96 to 1.02)	0.576	**1.02**	**(1.01 to 1.04)**	**0.007**
Health facilities									
Health centre versus none	1.13	(0.81 to 1.54)	0.467	1.12	(0.83 to 1.51)	0.462	0.98	(0.65 to 1.48)	0.922
Hospital versus none	1.16	(0.91 to 1.46)	0.218	0.87	(0.46 to 1.62)	0.656	**1.45**	**(1.04 to 2.03)**	**0.027**
Road connectivity									
Yes versus no	**1.41**	**(1.05 to 1.88)**	**0.021**	1.22	(0.82 to 1.8)	0.333	1.19	(0.71 to 1.98)	0.505
Geographical division									
Community versus countryside	1.19	(0.79 to 1.76)	0.395	0.81	(0.41 to 1.58)	0.53	1.01	(0.5 to 2.02)	0.977
Town versus countryside	1.008	(0.75 to 1.34)	0.955	1.08	(0.82 to 1.41)	0.562	0.98	(0.66 to 1.45)	0.921
City versus countryside	1.17	(0.91 to 1.48)	0.214	0.81	(0.41 to 1.55)	0.526	**1.46**	**(1.03 to 2.07)**	**0.034**
Urban closeness									
Distant versus urban	0.82	(0.61 to 1.1)	0.187	0.84	(0.56 to 1.26)	0.394	**0.62**	**(0.43 to 0.87)**	**0.006**
Periphery versus urban	1.31	(0.97 to 1.74)	0.076	1.43	(0.97 to 2.1)	0.067	0.88	(0.54 to 1.49)	0.591

*Adjusted by sex, environmental tabaco smoke, farm animals and bronchitis.

†Adjusted by household overcrowding, farm animals, bronchitis, and atopy.

‡Increase by point (range 0–10).

§Increase by 10 000 population.

¶increase by 1000 population /km^2^.

**increase by 10 percentual points.

### Comparisons between the urbanicity scale and other common measures of urbanicity

**Figure 3:**
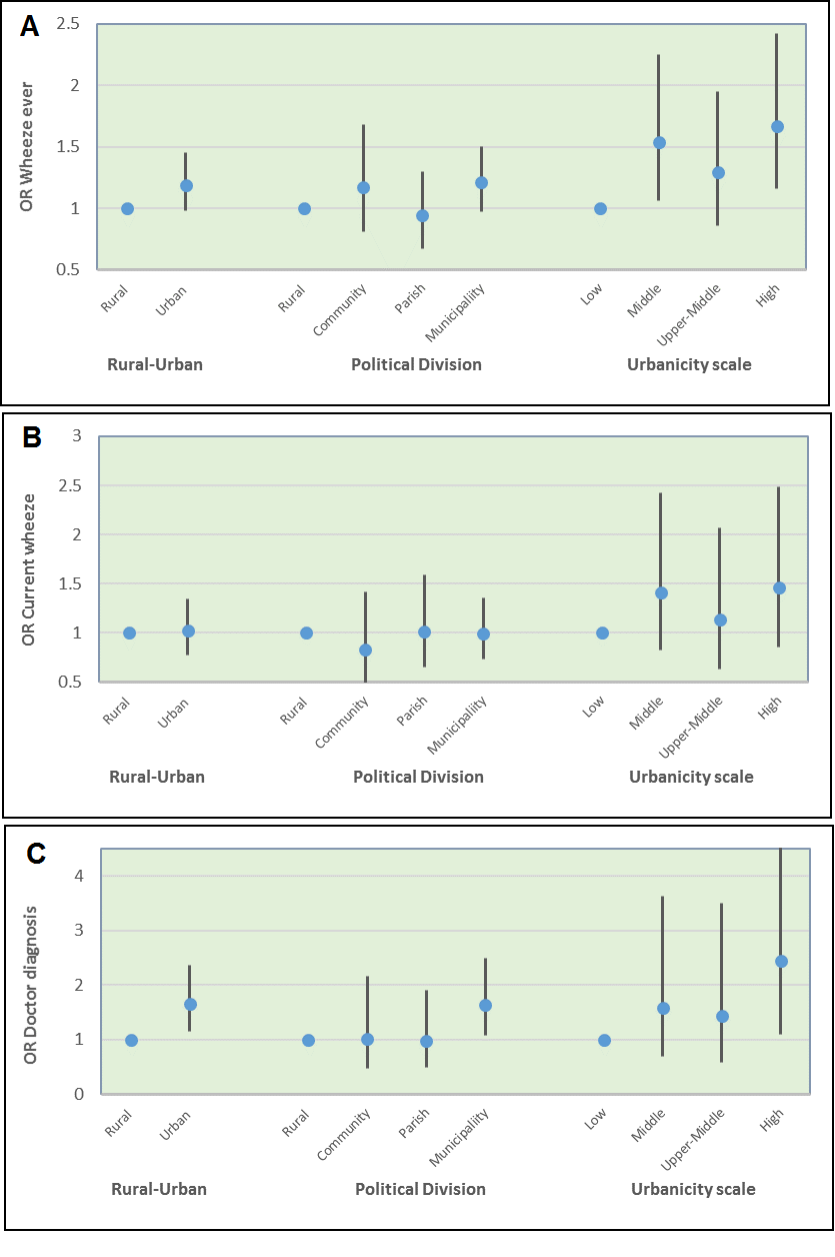
Comparison of the urbanicity measure with the urban–rural dichotomy and geopolitical division. (A) Wheeze ever. (B) Current wheeze. (C) Doctor diagnosis of asthma.

Comparisons between risks of the asthma using the three definitions by levels of urbanicity (four categories), urban–rural dichotomy and political divisions (four categories) are shown in figure 3. For wheeze ever, children living in high (OR=1.67, 95% CI 1.16 to 2.42, p=0.006) and middle (OR=1.54, 95% CI 1.06 to 2.25, p=0.025) urbanicity wards had a greater risk of asthma than those living in low urbanicity wards. Likewise, for doctor diagnosis, children living in high urbanicity wards (OR 2.44, 95% CI 1.10 to 5.41, p=0.028) had a greater risk of asthma compared with those living in low urbanicity wards.

### Univariate and multivariate analyses between levels of urbanicity (four categories), asthma definitions and other asthma risk factors

Table 5 shows a univariate and multivariate analyses between asthma definitions associated with the urbanicity score (wheezing ever and doctor diagnosis) and several risk factors for asthma. For wheeze ever, multivariate analyses showed that children living in an area with high level of urbanicity had 1.59-fold risk of wheeze (OR=1.59, 95% CI 1.07 to 2.36, p=0.023), history of maternal asthma had 1.96 fold risk of wheeze (OR=1.96, 95% CI 1.31 to 2.93, p=0.001), living in an overcrowded house had 1.25-fold risk of wheeze (OR=1.25, 95% CI 0.97 to 1.61, p=0.084), diagnosed with bronchitis had 2.62-fold risk of wheeze (OR=2.62, 95% CI 1.97 to 3.48, p<0.001) and diagnosed with bronchopneumonia had 3.67-fold greater risk of wheeze (OR=3.67, 95% CI 2.47 to 5.46, p<0.001). For doctor diagnosis, multivariate analyses showed that children living in an area with high level of urbanicity had 2.21-fold risk of wheeze (OR=1.25, 95% CI 0.92 to 5.32, p=0.075), history of maternal asthma had 4.78-fold risk of wheeze (OR=4.78, 95% CI 2.86 to 7.91, p<0.001), dog inside the house had 1.77-fold risk of wheeze (OR=1.77, 95% CI 1.19 to 2.64, p=0.005), diagnosed with bronchitis had 2.76-fold risk of wheeze (OR=2.76, 95% CI 1.78 to 4.28, p<0.001) and atopy had 3.52-fold risk of wheeze (OR=3.52, 95% CI 2.12 to 5.98, p<0.001).

**Table 5:** Associations between urbanicity scale and asthma definitions and other asthma risk factors

Indicators	Categories	Wheeze ever						Doctor diagnosis					
		Univariate			Multivariate			Univariate			Multivariate		
		OR	95% CI	P value	OR	95% CI	P value	OR	95% CI	P value	OR	95% CI	P value
Urbanicity scale	Middle versus low	1.54	(1.06 to 2.25)	0.025	1.43	(0.96 to 2.15)	0.082	1.58	(0.69 to 3.62)	0.277	1.47	(0.59 to 3.63)	0.408
	Upper middle versus low	1.29	(0.86 to 1.95)	0.221	1.01	(0.64 to 1.58)	0.974	1.43	(0.59 to 3.50)	0.431	0.96	(0.35 to 2.62)	0.934
	High versus low	1.67	(1.16 to 2.42)	0.006	1.59	(1.07 to 2.36)	0.023	2.44	(1.10 to 5.41)	0.028	2.21	(0.92 to 5.32)	0.075
Sex	Male versus female	1.12	(0.93 to 1.36)	0.242				1.16	(0.81 to 1.67)	0.409			
Maternal asthma	Yes versus no	2.10	(1.46 to 3.04)	<0.001	1.96	(1.31 to 2.93)	0.001	4.81	(3.01 to 7.68)	<0.001	4.78	(2.86 to 7.91)	<0.001
Environmental tabaco Smoke	Yes versus no	1.01	(0.76 to 1.34)	0.996				1.24	(0.76 to 2.05)	0.390			
Household overcrowding	Yes versus no	1.20	(0.94 to 1.52)	0.139	1.25	(0.97 to 1.61)	0.084	1.17	(0.76 to 1.81)	0.470			
Dog at home	Yes versus no	1.24	(1.02 to 1.50)	0.033				2.01	(1.40 to 2.90)	<0.001	1.77	(1.19 to 2.64)	0.005
Farm animals	Yes versus no	0.97	(0.78 to 1.19)	0.750				0.80	(0.55 to 1.17)	0.255			
Bronchitis	Yes versus no	2.83	(2.18 to 3.68)	<0.001	2.62	(1.97 to 3.48)	<0.001	3.80	(2.57 to 5.60)	<0.001	2.76	(1.78 to 4.28)	<0.001
Bronchopneumonia	Yes versus no	4.45	(3.05 to 6.49)	<0.001	3.67	(2.47 to 5.46)	<0.001	4.68	(2.95 to 7.41)	<0.001			
Atopy	Yes versus no	1.12	(0.85 to 147)	0.430				1.46	(0.92 to 2.32)	0.111	3.52	(2.12 to 5.98)	<0.001

## Discussion

In the present study, we generated an urbanicity score to understand better how the process of urbanisation may be associated with the prevalence of childhood asthma in transitional areas of an LMIC. Our data showed a wide variation in levels of urbanicity across census wards within the two districts included in our analysis with evidence that the prevalence of childhood asthma was associated with increasing levels of urbanicity. Further, our data revealed which of the indicators included within the urbanicity index, were more relevant as determinants of prevalence of asthma symptoms at 5 years of age. Our analysis illustrates the benefits of using a multidimensional measure of urbanicity to study the epidemiology of asthma, allowing us to understand better which components may be most relevant, compared with more traditional urban–rural dichotomies.

Our findings are consistent with previous studies observing a higher risk of asthma in urban environments. Such studies have tended to use simple dichotomies of urbanicity in which populations living in large cities have been compared with those residing in rural towns or communities.^20^ Several asthma studies have used more than two categories to represent different levels of urbanicity.^21–26^ For example, studies conducted in Chile, Mozambique and Palestine compared asthma prevalence between urban, sub-urban, semi-rural and rural populations,^22 23 27^ observing a lower prevalence of asthma in rural settlements that increased with increasing urbanisation. Others studies have evaluated the effects of urbanisation on asthma by comparing populations living in different cities with diverse levels of urban development,^21 28 29^ For example, a study conducted in Peru compared the prevalence of asthma between four geographically distinct sites with varying levels of urbanisation (urban vs semiurban vs rural) and observed a greater prevalence with increasing levels of urbanisation,^21^ In contrast to previous studies, we used a continuous measure of urbanicity to identify a wide variation in urbanicity levels within a geographically localised area in Ecuador, and were able to show that higher levels of urbanicity were associated with a greater risk of asthma symptoms. However, not all ‘urban’ environments in LMICs increase asthma risk, at least when defined using a simple urban–rural dichotomy: some studies observed a lower prevalence of asthma in urban areas or were unable to detect a difference in prevalence between urban and rural populations.^30 31^

There is no generally accepted definition for asthma in epidemiological studies. Most studies have used a single definition, generally recent wheeze or a doctor diagnosis, which may measure different phenotypes and severity of asthma. However, both definitions are subject to limitations. Doctor diagnosis in rural settings of LMICs is affected by access to healthcare and the diagnostic criteria of individual doctors. Self-reported wheezing is affected by recall bias and by differing understandings of wheeze symptoms of subjects from distinct socio-cultural backgrounds. Additionally, questions about wheeze prior to the age of 6 are considered an unreliable measure of asthma in children. In the present study we used three definitions for asthma—wheeze ever, current wheeze and doctor diagnosis—allowing us to explore better the associations between urbanicity indicators and a wider range of disease phenotypes that may be represented by these definitions. Although several associations were identified, the presence and magnitude of these associations varied by phenotype. For example, doctor diagnosis of asthma was associated with 14 of 18 urban indicators, much greater than for wheeze ever (3 indicators) and recent wheeze (2 indicators). Differences in access to healthcare between urban and rural populations could explain associations with doctor diagnosis. Populations living in cities or other urban settlements have greater access to healthcare compared with rural populations because of the larger number of doctors and health institutions present.^32^ Wheeze ever or current wheeze may be better definitions for asthma in populations with highly variable access to doctors. However, asthma defined as current wheeze, may be less useful for contextual indicators of urbanicity in cross-sectional analyses because of the restricted time during which symptoms must be present (ie, previous 12 months). Urban lifestyles and conditions may require years or even decades to affect human health outcomes.^19^

Some urban indicators appeared to be consistently associated with wheeze/asthma, irrespective of the definition used. Positive trends between indicators for the socioeconomic dimension of urbanism such as rates of adult secondary education, commercial activities, cement housing and home computer and satellite TV access were observed for more than one asthma definition. Indicators such as home computer and satellite TV access could be related to sedentarism within households. Low rates of physical activity and time spent indoors have been associated with asthma.^33 34^ The association between the proportion of cement-built houses within a census ward and asthma, might be explained by higher levels of humidity and mould exposure. In transitional areas, such houses are generally built without regulatory controls and often suffer from poor ventilation and high humidity. Several studies have observed associations between asthma and humidity within the house and history of respiratory infections.^35^ Our study did not find associations between the urbanicity score, urban indicators (except for electricity), and atopy. A possible explanation for this trend is that non-atopic asthma is the most common phenotype in Ecuador. Previous studies conducted in Ecuador and Brazil have estimated that 2.4%^36^ and 24.5%^37^ of wheeze cases are attributable to atopy, respectively, and are considerably lower than the 40.7% average reported in high-income countries.

As a categorical variable, our urbanicity scale identified differences in asthma prevalence (especially measured as wheeze ever and doctor diagnosis) between four differences levels of urbanicity, differences that were not detected by standard urban–rural and political-division approaches (figure 3). For example, middle urbanicity and high urbanicity were associated with a greater prevalence of asthma. However, the apparent greater prevalence of asthma observed in middle urbanicity level could be explained by ‘urban sprawl’—in which urban influences extend from the towns to adjacent rural areas such that rural populations living close to urban centres can experience similar social and environmental exposures to those living within the urban environment.^32^ Additionally, other factors such as urban poverty and lack of urban services in the peripheries of towns may be also responsible for these differences.^10 38^ At the same time, geographic indicators used in our analysis (urban closeness) showed that children residing in census wards close to these urban centres had a higher prevalence of asthma compared with children living in more distant wards.

Several epidemiological studies have used scale-based approaches to measure the effects of urbanicity on the risk of other non-communicable diseases.^12 39^ Of these, most have used community-level data to develop urbanicity scales although the urban indicators used tended to vary depending on study context, level of urbanisation, unit of analysis and availability of information. Some studies have applied methodologies with predefined scale algorithms to rank each indicator which when added was used to quantify level of urbanicity.^40 41^ Other studies have used more complex statistical procedures such as data reduction techniques to generate indexes or scales of urbanicity.^18 30^The score in the present analysis used 18 relevant urban indicators from publicly available data representing demographic, socioeconomic, infrastructure and geographical dimensions of the urbanisation process. Our urbanicity score was strongly associated with 17 of the 18 indicators (r>0.40) indicating good internal consistency.

The present analysis is subject to several limitations. The cross-sectional design does not allow us to determine the direction of the effects between urbanicity and asthma risk. Further, we did not consider potential effects of migration between areas of different urbanicity, a phenomenon that may be frequent in this study population. It is important to clarify that other relevant dimensions could be included in the analyses, for example, dimensions that represent urban lifestyles or urban social strain. In the case of our study, urban lifestyles and urban social strain indicators were not included because of these indicators are scarce in the national databases of LMICs. Although urban indicators are derived from the 2010 census, data on asthma outcomes were collected between 2013 and 2015. It is important to aware that our CATPCA model was built based on the information of 330 census wards, the entire study area of the cohort. However, only 157 wards (areas where the children were living) were used to evaluate the associations between asthma and urbanicity. This difference may explain the fact that some variables with high component loadings in the general model were not associated with asthma in the bivariate analyses. Urbanisation is a process that takes place over time and which involves a range of processes determined by cultural, historical, economic, and other dimensions that are difficult to define and measure. Inclusion of all such factors in any analysis is challenging. However, this project is the base for futures analyses involving migration studies and multilevel analysis evaluating associations between asthma prevalence and urbanisation using variables at census ward, household, and individual levels.

In conclusion, this study has used a multidimensional urbanicity approach to evaluate the associations between urbanisation and asthma prevalence. Our data provide evidence that even small-scale increases in urbanicity levels in rural populations may be associated with a higher prevalence of asthma. The present study also shows that the use of a multicomponent scale for measuring urbanicity can identify more clearly the relationship between the process of urbanisation and asthma and challenges also the prevailing use of urban–rural dichotomies in asthma research. We need to start thinking about more complex chains of causation in urban studies and asthma and we believe that such a multifactorial approach for asthma studies will provide novel insights into how urbanisation affects asthma prevalence.
