# Supplementary material for: Measuring urbanicity as a risk factor for childhood wheeze in a transitional area of coastal ecuador: a cross-sectional analysis
Source: BMJ Open Respir Res. 2020 Nov 30;7(1):e000679. doi: 10.1136/bmjresp-2020-000679 (PMC7705553; doi:10.1136/bmjresp-2020-000679)
Supplement: Supplementary data [file bmjresp-2020-000679supp001.pdf]

SUPPLEMENTARY FIGURES AND TABLES

Supplementary Figure SF1. Percentage of variance accounted by variables of each dimension.

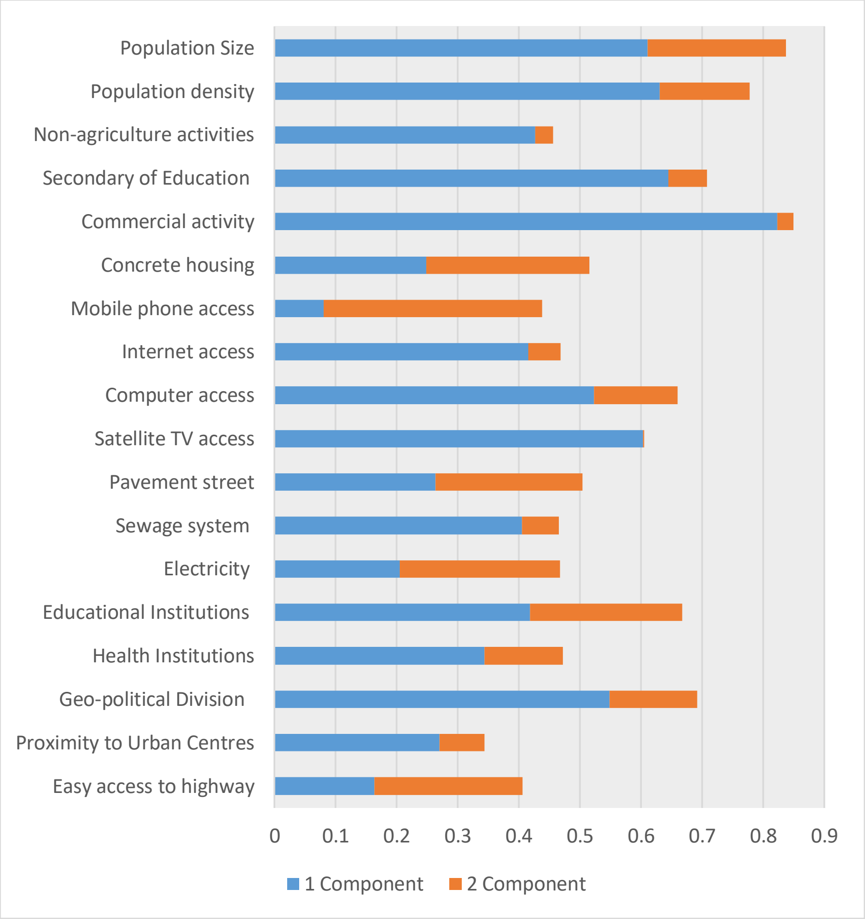

Supplementary Table 1. Categories and Quantifications derived from Categorical Principle Component Analysis.

**Population Size**			4296.85	1	3.114	78.95	1	2.945	27.08 - 27.74	5	1.785	30.51 - 30.85	2	1.881	65.38	1	1.097
**Category**	**Freq**	**Value**	4583.00 - 4587.76	2	3.114	82.28	1	3.439	27.84 - 28.00	3	1.785	31.62	1	1.881	66.67 - 66.94	3	1.097
36 - 226	98	-0.315	4864.89 - 4899.78	2	9.148	85.07	1	3.439	28.72 - 28.99	3	1.785	33.89	1	3.44	68.83 - 69.81	3	1.097
227 - 461	189	-0.231	**Non-agricultural activities**			86.22 - 86.31	2	5.432	30.29 - 30.58	2	1.785	34.58 - 34.68	2	3.44	72	1	1.097
468 - 682	25	0.688	**Category**	**Freq**	**Value**	95.7	1	5.432	31.03 - 31.33	2	1.785	37.86	1	3.44	72.50 - 74.19	3	1.097
706 - 922	7	1.288	0	1	-0.953	98.15	1	5.432	32.39	1	1.83	38.24 - 38.90	3	3.44	76.67 - 77.30	2	1.097
1142	1	1.367	3.85 - 4.76	5	-0.718	**Adult secondary education**			33.5	1	2.315	39.09	1	3.44	**Mobile phone access**		
2597 - 2767	2	2.14	5.08 - 6.90	15	-0.718	**Category**	**Freq**	**Value**	33.98	1	5.315	44.22	1	4.135	**Category**	**Freq**	**Value**
3080 - 3186	2	2.971	6.94 - 8.55	10	-0.718	0	1	-1.197	38.39	1	6.972	51.05	1	5.274	18.97	1	-2.49
3306	1	2.971	8.82 - 10.68	18	-0.718	0.81	1	-1.197	43.04	1	6.972	51.71	1	10.878	30.36	1	-2.49
3634	1	4.297	10.74 - 12.62	21	-0.605	1.2	1	-1.197	**Commercial activity**			**Cement housing**			33.33	2	-2.49
4726	1	4.942	12.63 - 14.49	19	-0.524	1.89 - 2.31	7	-1.197	**Category**	**Freq**	**Value**	**Category**	**Freq**	**Value**	35.00 - 35.14	2	-2.49
10169	1	6.015	14.58 - 16.39	19	-0.469	2.73 - 3.23	6	-1.17	0	23	-0.449	.00 - 1.08	24	-2.052	37.18 - 37.86	4	-2.226
29310 - 29356	2	9.225	16.51 - 18.33	19	-0.469	3.32 - 3.92	7	-0.991	.53 - .93	12	-0.449	1.56 - 3.16	14	-1.353	38.24	1	-1.971
**Population density**			18.75 - 20.18	14	-0.45	3.98 - 4.69	15	-0.899	1.10 - 1.87	25	-0.449	3.39 - 5.17	6	-1.199	39.76 - 40.00	3	-1.971
Category	Freq	Value	20.54 - 21.98	12	-0.45	4.76 - 5.44	13	-0.723	1.94 - 2.88	41	-0.449	5.88 - 6.90	4	-1.199	41.18	1	-1.971
1.49 - 9.02	36	-0.288	22.22 - 23.76	14	-0.351	5.47 - 6.15	17	-0.723	2.90 - 3.75	26	-0.429	7.41 - 9.09	13	-1.01	42.35 - 42.86	3	-1.971
9.09 - 98.97	248	-0.288	24.36 - 25.68	9	-0.351	6.25 - 6.94	18	-0.669	4.00 - 4.76	21	-0.429	9.33 - 10.94	6	-1.01	44.23	1	-1.655
107.34 - 175.27	7	0.152	26.09 - 27.59	9	-0.156	7.02 - 7.62	15	-0.661	4.84 - 5.71	24	-0.382	11.11 - 12.99	9	-1.01	47.23 - 47.73	4	-1.655
209.28 - 282.03	2	0.453	28.00 - 29.55	14	-0.156	7.73 - 8.40	17	-0.599	5.75 - 6.56	14	-0.382	13.21 - 15.00	18	-0.964	48.78	1	-1.655
474.79 - 558.14	2	0.453	30.00 - 31.51	15	-0.156	8.44 - 9.09	16	-0.593	6.73 - 7.58	12	-0.382	15.22 - 16.67	12	-0.41	50	1	-1.655
884.51	1	0.475	32.14 - 33.62	6	-0.15	9.19 - 9.92	16	-0.261	7.69 - 8.54	9	-0.252	17.24 - 18.82	9	-0.41	51.58	1	-1.655
956.23	1	0.475	34.01 - 35.36	10	0.166	10.00 - 10.67	8	-0.261	8.65 - 9.46	9	-0.252	19.61 - 20.75	9	-0.312	52.07 - 53.33	6	-1.655
1346.62 - 1384.59	2	0.672	35.59 - 37.44	6	0.166	10.67 - 11.29	14	-0.261	9.52 - 10.40	14	-0.015	21.05 - 22.77	12	-0.312	53.62 - 54.24	4	-1.655
1399.89	1	0.672	38.16 - 39.31	6	0.166	11.46 - 12.08	7	-0.261	10.57 - 11.36	9	0.104	23.44 - 24.64	7	-0.018	55.93 - 56.72	5	-0.703
1543.19	1	0.672	39.39 - 41.24	5	0.166	12.20 - 12.88	14	0.173	11.59 - 12.06	3	0.104	25.00 - 26.83	14	-0.018	57.45 - 57.89	6	-0.703
1600.85	1	0.672	41.28 - 42.86	12	0.262	12.90 - 13.61	16	0.173	12.50 - 12.88	9	0.104	27.06 - 28.85	6	-0.018	58.70 - 59.78	7	-0.703
1692.75	1	0.672	43.33 - 45.05	6	0.262	13.73 - 14.29	9	0.173	13.33 - 13.91	8	0.104	28.89 - 30.77	10	-0.018	60.22 - 60.53	6	-0.689
1831.26	1	0.672	45.28 - 46.81	9	0.262	14.49 - 15.10	12	0.173	14.34 - 14.96	5	0.104	30.95 - 32.56	13	0.2	61.18 - 62.07	7	-0.536
1948.75 - 1984.76	2	1.304	47.68 - 48.59	6	0.466	15.24 - 15.85	7	0.173	15.29 - 16.09	4	0.27	32.91 - 34.67	9	0.734	62.77 - 63.64	11	-0.536
2125.48	1	1.304	49.04 - 50.79	8	0.466	15.89 - 16.56	11	0.194	16.18 - 17.07	7	0.442	34.78 - 36.36	12	0.734	64.00 - 64.94	11	-0.536
2289.62	1	1.304	52.48	1	0.466	16.67 - 17.31	9	0.297	17.14 - 17.88	7	0.496	37.04 - 38.46	12	0.734	64.95 - 66.20	9	-0.536
2320.82 - 2325.26	2	1.304	52.83 - 54.55	6	0.466	17.53 - 17.91	6	0.537	18.06 - 18.78	4	0.496	39.53 - 40.54	5	0.734	66.28 - 67.44	11	-0.536
2414.12 - 2465.53	4	2.275	54.90 - 56.52	4	0.466	18.13 - 18.83	7	0.537	19.15 - 19.87	4	0.496	41.03 - 42.55	8	0.895	68.00 - 68.75	10	-0.536
2502.83	1	2.547	56.94 - 58.33	3	0.466	19.30 - 19.39	3	0.537	20.75	1	0.496	42.94 - 44.44	9	0.895	68.89 - 70.00	13	-0.536
2696.57	1	2.547	59.19 - 59.70	2	1.396	19.59 - 20.26	7	0.537	21.37 - 21.67	2	0.496	44.64 - 46.51	10	1.068	70.18 - 71.25	22	-0.536
2923.71	1	2.547	61.11 - 62.21	3	1.396	20.55 - 20.72	3	0.871	22.22 - 22.58	3	0.496	46.81 - 48.50	11	1.068	71.43 - 72.46	11	0.314
3053.99	1	2.547	62.86 - 63.21	4	1.396	21.13 - 21.58	8	0.871	23.20 - 23.49	3	0.836	48.68 - 50.50	14	1.068	72.73 - 73.91	17	0.314
3196.02	1	2.547	64.71	1	1.396	21.96 - 22.50	3	0.871	24.53 - 24.64	2	0.836	50.65 - 52.50	9	1.068	74.00 - 75.18	16	0.314
3260.98	1	2.547	66.85 - 67.46	2	2.945	22.65 - 22.96	4	0.871	24.71 - 25.30	4	0.836	52.63 - 54.26	5	1.068	75.32 - 76.47	13	0.472
3357.47	1	2.547	68.75 - 69.44	4	2.945	23.49 - 23.59	2	1.785	25.76	1	0.836	54.72 - 56.45	8	1.068	76.54 - 77.78	19	0.646
3541.13	1	2.547	71.82	1	2.945	24.20 - 24.66	4	1.785	26.81 - 27.14	4	0.836	56.79 - 57.14	3	1.097	78.02 - 79.09	15	0.646
3688.84	1	2.547	73.13	1	2.945	24.82 - 25.25	3	1.785	27.54 - 28.30	2	0.836	59.09 - 60.27	5	1.097	79.25 - 80.36	15	0.646
3773.54	1	2.547	74.6	1	2.945	25.7	1	1.785	28.57 - 28.87	3	0.836	60.53 - 62.35	7	1.097	80.41 - 81.61	16	0.874
4038.19	1	3.114	75.66 - 76.44	2	2.945	26.39 - 27.01	2	1.785	29.46	1	1.881	62.90 - 64.17	4	1.097	82.00 - 82.98	10	0.874

Continuation supplementary table 1

83.15 - 84.21	6	0.874	9.52	1	2.933	21.62	1	2.804	11.76 - 12.06	3	1.168	9.09	1	1.264	77.27 - 79.25	9	-0.324
84.38 - 85.53	6	1.18	10.28 - 10.34	2	2.933	23.08	1	2.804	12.50 - 13.79	5	1.168	9.38	1	1.264	79.59 - 81.63	8	-0.324
85.71 - 86.84	8	1.18	10.69	1	4.077	24.84	1	5.014	14.00 - 14.81	3	1.168	12.5	1	1.264	81.82 - 83.72	13	-0.208
87.18 - 87.50	6	1.18	11.76	1	4.077	27.94	1	5.483	15.56 - 16.82	4	1.168	15.49	1	1.264	84.04 - 85.98	24	-0.208
88.24 - 89.19	6	1.286	12.9	1	4.077	**Satellite TV access**			17.59 - 18.06	3	1.168	22.8	1	5.878	86.25 - 88.28	34	-0.171
89.58 - 90.70	3	1.286	13.04	1	4.077	**Category**	**Freq**	**Value**	19.98 - 20.00	3	1.876	25.99	1	5.878	88.52 - 90.70	33	0.631
90.91 - 91.67	3	1.286	13.33	1	4.077	0	165	-0.561	21.88 - 22.78	4	1.876	30.37	1	5.878	90.91 - 92.94	31	0.817
92.16 - 92.52	2	3.002	**Computer access**			.83 - 1.37	31	-0.263	23.81 - 24.14	2	1.876	35.17	1	5.878	93.12 - 95.12	32	0.817
93.33	1	3.002	**Category**	**Freq**	**Value**	1.45 - 2.13	27	-0.263	25.00 - 25.71	3	1.876	41.68	1	5.878	95.29 - 97.06	27	1.149
95.74	1	3.002	0	101	-0.887	2.15 - 2.86	14	0.373	27.42 - 27.63	2	1.876	51.33	1	5.878	97.67 - 99.01	12	1.398
96.97	1	3.002	.70 - .87	2	-0.663	3.03 - 3.61	18	0.373	28.17	1	1.876	61.17	1	5.878	100	10	1.398
97.22	1	3.002	.91 - 1.33	15	-0.659	3.65 - 4.35	7	0.487	29.63 - 31.03	4	1.876	74.03	1	5.878	**Educational institutions**		
**Internet access**			1.37 - 1.79	17	-0.457	4.38 - 5.08	12	0.487	31.43	1	1.876	**Electricity**			**Category**	**Freq**	**Value**
**Category**	**Freq**	**Value**	1.89 - 2.20	16	-0.231	5.17 - 5.63	4	0.487	32.91 - 34.21	2	1.876	**Category**	**Freq**	**Value**	0	83	-0.2
0	139	-0.686	2.30 - 2.70	18	-0.231	5.88 - 6.52	8	0.763	35.48	1	1.876	0	2	-2.48	1	145	-0.2
.66 - .73	3	-0.686	2.82 - 3.13	17	-0.231	6.82 - 7.14	3	0.763	36.00 - 37.31	2	1.876	1.35 - 2.22	3	-2.48	2	61	-0.069
.88 - 1.00	8	-0.686	3.23 - 3.64	17	-0.182	7.46 - 7.81	3	0.763	37.50 - 38.46	3	1.876	2.90 - 3.57	2	-2.33	3	22	0.037
1.03 - 1.26	24	-0.273	3.65 - 4.00	11	0.091	8.41 - 8.77	4	0.763	39.53	1	1.899	6.67	1	-2.33	4	11	1.072
1.27 - 1.49	12	-0.259	4.11 - 4.55	15	0.263	8.82 - 9.52	7	0.763	40.58	1	1.899	7.84 - 8.51	2	-2.33	5	3	1.072
1.52 - 1.75	20	-0.092	4.60 - 4.92	5	0.263	9.68 - 10.14	5	1.679	42.06 - 43.33	5	1.899	15.05	1	-2.33	7	1	1.072
1.79 - 2.00	7	-0.092	5.05 - 5.48	12	0.263	10.39 - 10.91	2	1.679	44.78	1	1.899	17.39 - 17.44	2	-2.3	8	1	5.607
2.04 - 2.17	8	-0.092	5.62 - 5.83	5	0.263	12.00 - 12.43	4	1.679	45.61	1	1.899	22.22 - 22.89	3	-2.3	9	1	6.683
2.27 - 2.50	14	-0.086	5.97 - 6.39	9	0.529	12.77	1	1.679	47.41 - 48.08	2	1.899	25	1	-2.3	20	1	7.697
2.53 - 2.74	10	0.208	6.45 - 6.82	8	0.529	16.68	1	1.705	48.65 - 48.98	3	1.899	25.33 - 27.50	2	-2.3	21	1	12.959
2.78 - 2.92	4	0.208	6.90 - 7.14	5	0.529	17.28	1	1.705	51.46 - 51.76	2	1.899	29.55	1	-2.25	**Health institutions**		
3.03 - 3.23	13	0.6	7.44 - 7.66	4	0.89	18.42 - 18.82	2	1.705	56.53	1	1.899	32.81 - 34.15	2	-2.25	**Category**	**Freq**	**Value**
3.28 - 3.45	9	0.6	7.81 - 8.24	6	1.02	19.70 - 19.80	2	1.927	58.59	1	1.899	34.62 - 35.80	2	-2.25	None	305	-0.277
3.51 - 3.70	6	0.6	8.33 - 8.70	7	1.183	23.21	1	1.927	60	1	1.899	36.67 - 38.46	3	-1.68	Health centre	23	3.109
3.77 - 3.97	4	0.6	8.82 - 8.93	3	1.183	24.44	1	1.927	65.22	1	1.899	38.96 - 40.91	5	-1.24	Hospital	2	6.494
4.00 - 4.05	3	0.6	9.24 - 9.52	5	1.183	29.6	1	1.927	66.67	1	1.899	42.06	1	-1.24	**Close access to highway**		
4.29 - 4.48	7	0.6	9.68 - 10.02	4	1.183	32.18	1	1.927	71.11	1	1.899	44.12	1	-1.24	**Category**	**Freq**	**Value**
4.55	2	0.6	10.34 - 10.53	3	1.183	39.13	1	1.927	71.79	1	1.899	45.68 - 47.83	3	-1.24	No	221	-0.702
4.88 - 4.92	2	0.6	10.61 - 10.94	3	1.94	41.34	1	4.017	76.60 - 77.50	2	2.839	49.00 - 50.00	2	-1.24	Yes	109	1.424
5.00 - 5.10	2	0.672	11.54 - 11.91	2	2.282	45.95	1	4.017	**Sewage system**			50.60 - 52.27	5	-1.24	**Geo-political division**		
5.26 - 5.41	6	1.914	12.06	1	2.282	52.49	1	8.541	**Category**	**Freq**	**Value**	53.03 - 53.33	4	-1.14	**Category**	**Freq**	**Value**
5.49 - 5.62	3	1.914	12.5	1	2.282	66.18	1	8.541	.00 - .66	238	-0.36	55.17 - 55.70	2	-1.14	Rural area	292	-0.324
5.77	1	1.914	12.91 - 13.16	2	2.305	**Paved streets**			.67 - 1.35	20	0.254	57.14 - 58.33	4	-1.14	Community	30	1.877
6.00 - 6.19	4	2.138	13.54 - 13.64	2	2.305	**Category**	**Freq**	**Value**	1.41 - 2.08	22	0.366	59.38 - 60.53	2	-0.83	Town	5	3.978
6.25 - 6.38	3	2.138	14.57	1	2.432	.00 - 1.30	178	-0.734	2.13 - 2.72	17	0.497	61.54 - 62.71	7	-0.83	City	3	6.179
7.14 - 7.23	2	2.138	15.15	1	2.432	1.37 - 2.70	30	-0.253	2.86 - 3.45	9	0.497	65.33	1	-0.83	**Proximity to urban centres**		
7.49 - 7.69	2	2.933	15.19 - 15.52	2	2.432	2.94 - 4.44	18	0.113	3.61 - 3.95	6	0.497	66.28 - 67.44	6	-0.75	**Category**	**Freq**	**Value**
8.11	1	2.933	16	1	2.432	4.49 - 6.00	15	0.113	4.65	1	0.497	68.42 - 70.11	7	-0.32	Far from the city	271	-0.452
8.82	1	2.933	17.54 - 17.78	3	2.432	6.10 - 7.58	7	0.213	5.13 - 5.38	3	0.497	70.49 - 72.17	6	-0.32	Close to the city	55	1.914
9	1	2.933	18.79	1	2.804	7.81 - 8.89	3	0.918	5.98	1	1.264	73.44 - 74.51	4	-0.32	City	4	4.279
9.3	1	2.933	19.57	1	2.804	9.32 - 10.71	8	0.918	7.67	1	1.264	75.00 - 77.01	10	-0.32			

Supplementary Table 2. Bivariate logistic regression between urbanicity score, urban variables and atopy

	SPT		
Indicators	OR	95% CI	p
**Urbanicity Score [A]**	**0.94**	**(0.90-0.99)**	**0.014**
**Population Size [B]**	**0.80**	**(0.68-0.92)**	**0.002**
**Population density [C]**	**0.91**	**(0.84-0.98**	**0.018**
**Non-Agriculture activities [D]**	0.94	(0.87-1.003)	0.064
**Secondary Education [D]**	0.93	(0.74-1.12)	0.479
**Commercial activities [D]**	**0.91**	**(0.82-0.99)**	**0.046**
**Concrete housing [D]**	0.96	(0.86-1.07)	0.464
**Mobile phone access [D]**	0.99	(0.79-1.21)	0.995
**Internet access [D]**	**0.94**	**(0.90-0.98)**	**0.015**
**Computer access [D]**	0.97	(0.68-1.26)	0.830
**Satellite TV access [D]**	0.94	(0.84-1.05)	0.292
**Pavement street [D]**	1.04	(0.96-1.13)	0.325
**Sewage system [D]**	0.89	(0.76-1.02)	0.109
**Electricity [D]**	**1.15**	**(1.0-1.31)**	**0.046**
**Educational Institutions**	**0.97**	**(0.95-0.99)**	**0.002**
**Health facilities**			
**Health centre vs None**	0.82	(0.51-1.34)	0.437
**Hospital vs None**	**0.60**	**(0.16-0.25)**	**0.001**
**Road connectivity**			
**Yes vs No**	0.88	(0.61-1.26)	0.478
**Geographical division**			
**Community vs Countryside**	0.88	(0.53-1.48)	0.652
**Town vs Countryside**	**0.59**	**(0.36-0.98)**	**0.042**
**City vs Countryside**	**0.58**	**(0.43-0.78)**	**<0.001**
**Urban closeness**			
**Distant vs Urban**	**0.66**	**(0.45-0.96)**	**0.030**
**Periphery vs Urban**	**0.51**	**(0.38-0.69)**	**<0.001**

A= increase by point (Range 0-10); B= increase by 10,000 population;
C= increase by 1000 population /km$^{2)}$; D= increase by 10 percentual points.
SPT= skin prick test
